# Global Analysis of *Plasmodium falciparum* Dihydropteroate Synthase Variants Associated with Sulfadoxine Resistance Reveals Variant Distribution and Mechanisms of Resistance: A Computational-Based Study

**DOI:** 10.3390/molecules28010145

**Published:** 2022-12-24

**Authors:** Rita Afriyie Boateng, James L. Myers-Hansen, Nigel N. O. Dolling, Benedicta A. Mensah, Elia Brodsky, Mohit Mazumder, Anita Ghansah

**Affiliations:** 1 Noguchi Memorial Institute for Medical Research, College of Health Sciences, University of Ghana, Legon, Accra P.O. Box LG 581, Ghana; 2 Pine Biotech, Inc., 1441 Canal St., New Orleans, LA 70112, USA

**Keywords:** *Pf*dhps, sulfadoxine resistance, malaria, molecular dynamics simulations, molecular docking, haplotype, mutations

## Abstract

The continual rise in sulfadoxine (SDX) resistance affects the therapeutic efficacy of sulfadoxine-pyrimethamine; therefore, careful monitoring will help guide its prolonged usage. Mutations in *Plasmodium falciparum* dihydropteroate synthase (*Pf*dhps) are being surveilled, based on their link with SDX resistance. However, there is a lack of continuous analyses and data on the potential effect of molecular markers on the *Pf*dhps structure and function. This study explored single-nucleotide polymorphisms (SNPs) in *Pf*dhps that were isolated in Africa and other countries, highlighting the regional distribution and its link with structure. In total, 6336 genomic sequences from 13 countries were subjected to SNPs, haplotypes, and structure-based analyses. The SNP analysis revealed that the key SDX resistance marker, A437G, was nearing fixation in all countries, peaking in Malawi. The mutation A613S was rare except in isolates from the Democratic Republic of Congo and Malawi. Molecular docking revealed a general loss of interactions when comparing mutant proteins to the wild-type protein. During MD simulations, SDX was released from the active site in mutants A581G and A613S before the end of run-time, whereas an unstable binding of SDX to mutant A613S and haplotype A437A/A581G/A613S was observed. Conformational changes in mutant A581G and the haplotypes A581G/A613S, A437G/A581G, and A437G/A581G/A613S were seen. The radius of gyration revealed an unfolding behavior for the A613S, K540E/A581G, and A437G/A581G systems. Overall, tracking such mutations by the continuous analysis of *Pf*dhps SNPs is encouraged. SNPs on the *Pf*dhps structure may cause protein–drug function loss, which could affect the applicability of SDX in preventing malaria in pregnant women and children.

## 1. Introduction

Sub-Saharan Africa remains a major hub for malaria, with high morbidity and mortality, and children under the age of five and pregnant women are the most vulnerable populations [1]. Global efforts toward eliminating malaria are underway but have been hindered by increased drug resistance [1,2]. Monitoring the spread of drug-resistant parasites is paramount to steering control strategies.

One crucial antimalarial therapy against which resistance in the parasite population in malaria-endemic regions has rapidly developed is sulfadoxine–pyrimethamine (SP) [3,4]. As a result, SP is no longer a recommended treatment for uncomplicated malaria in sub-Saharan African countries and was replaced by the World Health Organization’s (WHO) proposed artemisinin-based combination therapy (ACT) [5]. Currently, however, SP represents the cornerstone therapy for the intermittent preventive treatment of malaria in pregnant women and children (IPTp/c) [6] across malaria-endemic regions [7]. Additionally, therapy based on a combination of SP and amodiaquine is used for seasonal malaria chemoprophylaxis (SMC) in Africa [8]. Although SP efficacy is gradually decreasing across Africa, there are variations in SP-resistance levels within Africa [7,9,10]; thus, SP could soon be compromised. Therefore, there is an urgent need to intensify the surveillance of SP resistance. This should involve mapping out the current geographical distribution and spread to assess both the evolution of SP resistance and the continued use of SP as a malaria control intervention. The impact of key mutations on the protein structure and, potentially, its function will provide insights into the mechanisms underlining SP resistance, particularly across Africa.

**Figure 1:**
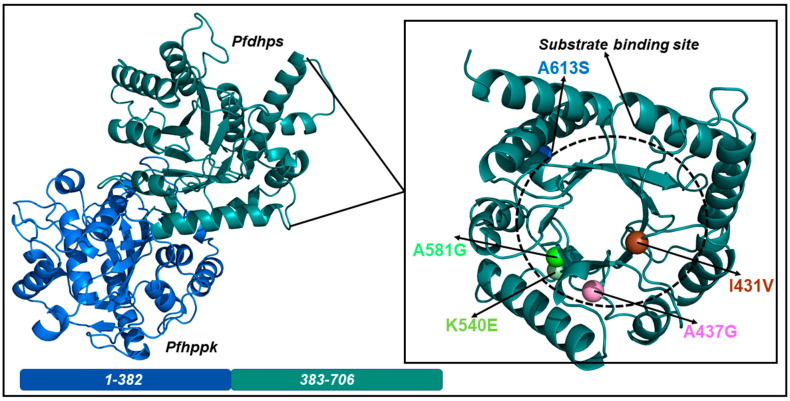
Cartoon representation of the structural components of *Pf*dhps–*Pf*hppk. The amino acid ranges for both proteins are indicated in bars, with the *Pf*dhps segment highlighted in deep teal and the *Pf*hppk segment in blue. Mutations occurring in *Pf*dhps are indicated by spheres in the right panel, which is a magnification of the region indicated on the structure to the left. The image was generated using the PyMOL visualizer.

SP is a combination therapy comprising sulfadoxine (SDX) and pyrimethamine (PYR), which act in synergy to inhibit *Plasmodium falciparum* folate synthesis. Pyrimethamine inhibits *P. falciparum* dihydrofolate reductase (*Pf*dhfr), whereas SDX targets *P. falciparum* dihydropteroate synthase (*Pf*dhps), an enzyme upstream of *Pf*dhfr in the folate synthesis pathway. Our understanding of the effect of *Pf*dhps mutations on the binding of SDX is highly dependent on the availability of a complete protein structure. *Pf*dhps is a bifunctional enzyme that catalyzes the conversion of 6-hydroxymethyl-7,8-dihydropterin pyrophosphate (DHPP) to 7,8 dihydropteroate following the addition of p-aminobenzoic acid (pABA) during *P. falciparum* folate biosynthesis. *Pf*dhps is 323 amino acids long and folds into a triosephosphate isomerase (TIM) barrel single-domain protein (Figure 1). The protein features a well-structured eight-stranded core of parallel *β-sheets* surrounded by peripheral α-helices. The active site of the protein is a highly flexible tunnel formed by the core *β-sheets*, flanked by loops. Research by Chitnumsub et al., in 2020, indicated that SDX binds at the active site, to interact with crucial catalytic residues [11]. When bound, SDX functions by competing with pABA, the substrate of *Pf*dhps, thereby inhibiting the conversion of DHPP to 7,8 dihydropteroate. This leads to the killing of the *P. falciparum* parasite by preventing the downstream synthesis of tetrahydrofolate, the necessary precursor for DNA synthesis. Additionally, drug activity can also be achieved via the conversion of DHPP to pterin-sulfa dead-end metabolic products [12].

Resistance to SP is linked to the accumulation of point mutations at multiple sites in both *Pf*dhfr and *Pf*dhps [13]. Thus far, eight resistance mutations (I431V, S436A/F, A437G, K540E, A581G, and A613S/T) in *Pf*dhps have been reported [14,15,16,17]. These mutations, together with the *Pf*dhfr mutations, confer resistance to the SP drug combination. Although other factors, such as the folate salvage capability of the parasite and host factors, have been established as key factors, the attribution of SDX resistance to *Pf*dhps has been widely validated via surrogate marker experiments [18,19]. The mutants A581G and A613S, which result in high-resistance phenotypes, have been detected at low frequencies in certain African countries (Ghana, Niger, Tanzania) [20,21,22]; however, comprehensive data for Central and West Africa remain scarce [14,21]. Intrinsically, a super-resistance genotype carrying A581G or A613S mutations, together with other virulent mutations, may cause high-level resistance to SDX. Nonetheless, these mutations are still emerging, especially in West Africa. There is, therefore, the need to map SP resistance to gain an insight into how widespread these mutations are in Ghana and also across Africa. Analysis of the initial data from Ghanaian parasite isolates has revealed that the prevalence of A581G and A613S mutations in the forest and coastal regions increased (<20%) over a 4-year surveillance period [23].

In this study, we investigated the prevalence and potential impact of drug-resistant *Pf*dhps mutations in Ghana and other African countries, Southeast Asia, and South America, using both genomic and protein structure-based approaches. The prevalence of mutations and key haplotypes at the genomic level were estimated. The 3D structure and features of the wild-type (WT) and mutant proteins were generated using homology modeling and per-residue energy-based approaches. The impact of mutations on SDX binding was revealed through molecular docking and molecular dynamics simulations. Overall, the detected mutant genotypes and haplotypes, which were observed at high frequencies, may have implications for the continued deployment of SP for IPTp/c, for the prevention of malaria in pregnant women and in children. Most importantly, a haplotype, i.e., A437G/A581G/A613S (implicated in conferring resistance) was identified in West Africa and Central Africa. Analysis of the protein structure and function revealed various crucial mechanisms of SDX resistance, which could be used to inform the basic rationale for novel drug discovery approaches.

## 2. Results

### 2.1. Prevalence of Pfdhps Mutations

A total of 6336 *P. falciparum* genomes representing malaria-endemic regions, i.e., Eastern Africa (EAF), Central Africa (CAF), Western Africa (WAF), Southeast Asia (SEA), and South America (SAM), were extracted from MalariaGEN Pf3k release 6 [24] and analyzed for the presence of *Pf*dhps mutations (Table S1 from the Supplementary Materials). The samples from Ghana included archived data collected from 2014 to 2017 [23]. The number of samples that had data available for the key *Pf*dhps mutations, A437G, K540E/N/Y, and A581G, were 5996, 5923, and 5955, respectively. Similarly, 5964 and 5846 samples had data for *Pf*dhps, i.e., A613S, and for the haplotype construction, respectively. The overall prevalence of *Pf*dhps mutations in each country is shown in Table 1. From the resultant data, seven significant mutations (greater than 1% of the population) were observed (A437G, K540E/N, A581G, A613S/T, and I431V).

The key SDX resistance marker, A437G, was nearing fixation in all studied countries, but a significant peak was observed in Malawi (100%) (Table 1). All the SNPs, apart from A437G, were identified to carry both WT and mutant alleles in varying rates of prevalence in the studied sample set. Two different alleles were seen to affect the codons 540 (K540E and K540N) and 613 (A613S and A613T), along with the WT. Interestingly, the rear K540N allele was seen in isolates from Southeast Asia (Thailand; 6.88%, Cambodia; 3.37%, Vietnam; 4.35%) and in one isolate each in Ghana (WAF) and Cameroon (CAF), but not in the EAF region. Moreover, the A613T mutant was only observed in Thailand (0.42%), Cambodia (0.53%), and Kenya (2.38%). With the exception of the Democratic Republic (DR) of Congo and Malawi, where the mutant A613S was absent in the analyzed parasite isolates, the mutant showed varying prevalence across the remaining locations.

Furthermore, the mutants K540E and A581G, which complete the full (quintuple) and super (sextuple)-SP-resistant haplotypes with *Pf*dhfr/*Pf*dhps, i.e., N51IC59RS108N A437G, were higher in the EAF and SEA regions. The prevalence was 87.1–99.6% and 37.5–91.5% for K540E, and 0.8–28.8% and 14.6–81.6% for A581G in the EAF and SEA regions, respectively. Conversely, the prevalence for A613S remained comparable in all countries. There were significant differences in the prevalence of each of the four SNPs across the regions: A437G (χ^2^ = 1085.6, *p* < 0.001), K540E/N/Y (χ^2^ = 5600.5, *p <* 0.001), A581G (χ^2^ = 2680, *p <* 0.001), and A613S/T (χ^2^ = 624.2, *p* < 0.001). Similarly, all four point mutations showed significant differences in prevalence in both the East African and Southeast Asian regions: A437G (χ^2^ = 24.4, *p <* 0.001) and (χ^2^ = 102.4, *p <* 0.001), K540E/N/Y (χ^2^ = 30.9, *p <* 0.001) and (χ^2^ = 867.5, *p <* 0.001), A581G (χ^2^ = 90.2, *p <* 0.001) and (χ^2^ = 494.1, *p* < 0.001), A613S/T (χ^2^ = 17.7, *p* < 0.001) and (χ^2^ = 300.4, *p <* 0.001), respectively. However, in Western and Central African regions, all but K540E/N/Y (χ^2^ = 5.97, *p* = 0.202) and A437G (χ^2^ = 0.35, *p* = 0.554) exhibited a significant difference in prevalence. In addition, for the South American region, only K540E/N/Y (χ^2^ = 2.57, *p* = 0.109) and A613S/T (χ^2^ = 1.22, *p* = 0.270) did not exhibit a significant difference. In addition, the I431V mutant allele was only observed in Cameroon (19%) and Ghana (1.4%).

### 2.2. Haplotype Frequencies

The frequency of key haplotypes is shown in Table 2. In general, 17 different *Pf*dhps haplotypes were identified. Of these, 9 haplotypes each were found in the three African regions (WAF, CAF, and EAF), while 5 and 14 haplotypes were found in South America and Southeast Asia, respectively. There were significant differences in the frequency of haplotypes across all countries (χ^2^ = 10736, *p* < 0.001). In addition, across individual regions, there were significant differences in the frequency of haplotypes in each region: WAF (χ^2^ = 434.0, *p* < 0.001), CAF (χ^2^ = 142.7, *p* < 0.001), EAF (χ^2^ = 162.3, *p* < 0.001), and SEA (χ^2^ = 1797.6, *p* < 0.001); however, in the SAM region, there was no significant difference in haplotype frequency (χ^2^ = 9.20, *p* = 0.053).

Furthermore, 8 of the 17 haplotypes showed relatively high frequencies (Table 2); these haplotypes consisted of two single (AKAS and GKAA), four double (AKGS, GEAA, GKGA, and GKAS), and one triple (GKGS) mutant haplotypes. The single mutation A518G, occurring on haplotype GKAA, exhibited the highest prevalence across all regions (range 0.0–84.7%). Single and double mutants (GKAA and GKAS) were present in all five regions, while another double mutant (GEAA) was present in all regions except SAM. In addition, the double mutant GKGA was found in the CAF, SEA, and SAM regions but not in WAF or EAF. The Central African region alone harbored the double mutant AKGS, while the WAF and EAF regions contained the single mutant AKAS. Interestingly, the triple mutant haplotype, GKGS, was only prevalent in Western and Central Africa.

### 2.3. Establishment of the Complete Protein Structure of Wild-Type and Mutant Pfdhps

**Figure 2:**
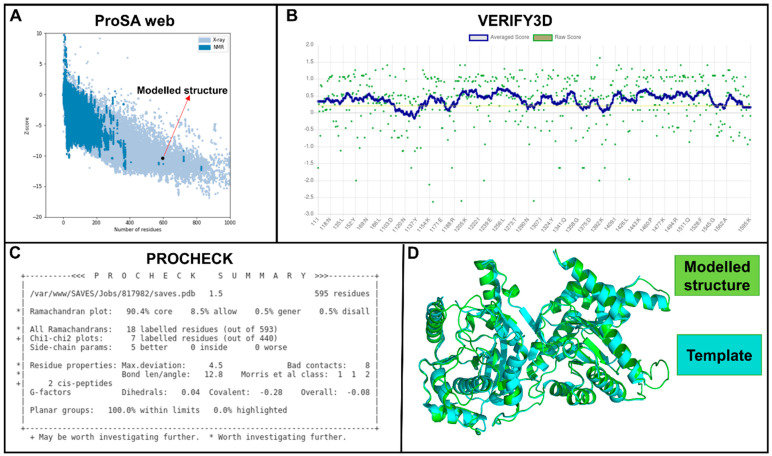
Evaluation scores of the modeled structures of WT *Pf*dhps for evaluation, performed via ProSA, Verify3D, PROCHECK, and PDB metrics. (**A**) ProSA results, showing that the modeled structure is comparable to X-ray structures of a similar size; (**B**) Verify3D data, indicating that all classified residues (alpha, beta, loop, polar, nonpolar etc.) are within the accepted window and environment; (**C**) Ramachandran plot by PROCHECK, showing that >90% of residues have the correct stereochemical structure; and (**D**) a superimposed image of the modeled and template structures (RMSD = 0.19 Å).

Prior to modeling, an appropriate template was selected (PDB ID: 6JWX) and assessed using ProSA [25], Verify3D [26], PROCHECK [27], and PDB metrics [28]. From these data, the template showed a resolution of 2.50 nm, indicating a clear density profile. One hundred models were generated, and the top three were evaluated in a similar procedure to that used for the template. From the results, ProSA predicted a Z-score of −10.33 (Figure 2A), indicating that the modeled structure is comparable to experimentally determined X-ray structures in PDB. The structure assessment by VERIFY3D indicated that most residues (85.5%) have averaged 3D–1D scores ≥0.2 (Figure 2B). Additionally, stereochemical checks via the PROCHECK tool revealed that 90.4% of *Pf*dhps residues fell within the most favorable regions (Figure 2C), implying an acceptable range, i.e., that of a good-quality structure.

### 2.4. Physiochemical and Structural Properties between Wild-Type and Mutant Proteins

Table 3 shows data for the key properties of the identified mutations. Overall, several types of changes in amino acid properties are observed. The mutations I431V, A437G, and A581G registered a change from a large hydrophobic residue (A = 89.1 Da, I = 131.2 Da) to a smaller hydrophobic residue (G = 75.1 Da, V = 117.1 Da). Interestingly, the mapping of mutations to structures indicates that A581G occupies the active site of a crucial loop structure that is in close proximity to the widespread resistance markers, A437G and K540E. In addition, I431V lies within the buried *β-sheets* that form the active site tunnel. K540E and A613S represent a change from a relatively small hydrophobic residue (A = 89.1 Da, K = 146.2 Da) to a polar residue (S = 105.1 Da, E = 147.1 Da). Furthermore, mutant A613S occurs on an alpha-helical structure that is distant from the active site of the protein, whereas K540E is near the active site.

The *DynaMut* tool was employed to deduce the effect of mutations on the structure. From the results, the mutations and haplotypes of I431V, A437G, K540E, A581G, A613S, A437G/A581G, and A437G/A581G/A613S are predicted to destabilize regions of the active site (entropy score = 0.31 to 0.66). On the other hand, the A437G/A613S haplotype is predicted to stabilize one region in the active site (entropy score = −0.06).

### 2.5. Evaluation of the Effect of Mutations on Sulfadoxine Binding

**Figure 3:**
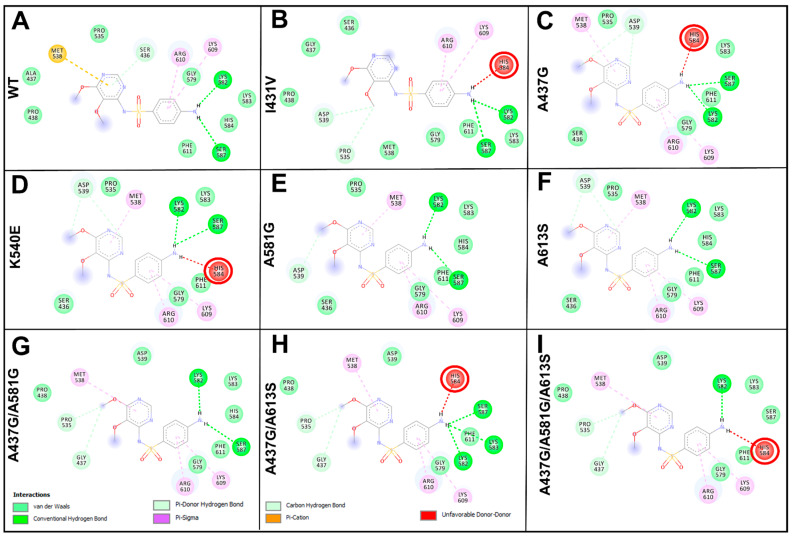
Molecular interaction fingerprint of SDX at the binding site of *Pf*dhps WT and the mutant proteins. Interaction fingerprints were generated using the DS visualizer. Hydrogen interactions are indicated in green. Unfavorable bonds are shown in red. (**A**) SDX interaction with the WT protein. (Panels (**B**–**F**)) SDX interacting with the single mutants I431V, A437G, K540E, A581G, and A613S, respectively. (Panels (**G**–**I**)) Interactions with the double mutants A437G/A581G and A437G/A613S and the triple mutant A437G/A581G/A613S, respectively. The pose/mode 4 in each docking cluster is shown.

Molecular docking was performed to fully explore the impact of mutations on SDX binding. This approach represents an important tool for studying protein–ligand interactions. Overall, single mutants (I431V, A437G, K540E, A581G, and A613S) and haplotypes composed of double (A437G/A581G, A437G/A613S, and K540E/A581G) and triple (A437G/A581G/A613S) mutations were subjected to docking using AutoDock Vina [29]. The changes to the binding energy scores varied across mutants, with affinities both increasing (A437G, −9.4; A613S, −9.4 kcal/mol) and decreasing (I431V, −6.2; K540E, −8.9; A437G/A581G, −8.9; K540E/A581G, −8.9; A437G/A581G/A613S, −7.8 kcal/mol) compared with WT (−9.3 kcal/mol). The mutant A581G (−9.3 kcal/mol) exhibited a similar binding affinity to that of the WT. Regarding molecular interactions, a general loss of molecular interactions was established in mutant proteins relative to the WT (Figure 3). SDX participated in three H-bonding interactions with Lys582, Ser436, and Ser587 in the WT protein. By comparison, two of these three residues in all mutants were found to participate in H-bonding interactions with SDX, i.e., all of them, with the exception of Ala436. In the presence of all mutants, the H-bonding interaction with Ala436 was replaced with a hydrophobic interaction. With regard to mutant I431V, A437G, and K540E and haplotype A581G/A613S and A437G/A581G/A613S, SDX exhibited an unfavorable interaction with the residue His584. Additionally, the complete loss of the pi–cation bond with Met538 was observed across all the mutants and haplotypes except A581G, A613S and A437G/A581G.

### 2.6. The Molecular Dynamics of SDX in Wild-Type and Mutant Dhps Binding Sites

**Figure 4:**
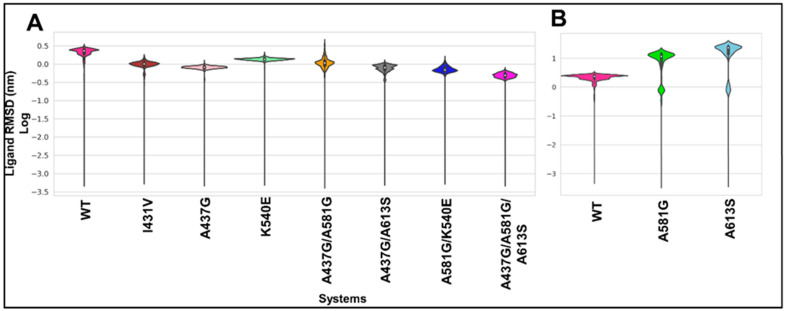
Kernel distribution plot showing ligand RMSD log scores occurring in WT and mutant proteins. The log of the RMSD values was calculated to the base e, where base e indicated the constant value, with an approximate value of 2.718282. The white dots represent the median, whereas the thick black bars in the centers illustrate the interquartile range. (**A**) WT (pink) and mutant systems where SDX was retained during the 150 ns simulation. (**B**) Mutant proteins where SDX was released before the simulation run time, compared to the WT (pink). The same WT system is represented on each panel but is shown on a different scale.

Molecular dynamics simulations were performed for a period of 150 ns. After the removal of all periodic boundary conditions (PBC), post-docking analyses were performed. The ligand root mean square deviation (RMSD) was estimated to evaluate the dynamics of SDX at the binding site of the WT and mutant proteins. Overall, SDX behaved differently toward all mutants in comparison to WT (Figure 4 and Figure S1 in the Supplementary Materials). In the WT protein, a bimodal conformational distribution was observed with a median RMSD of approximately 2.30 nm (log value = 0.4). Regarding the mutant A581G and A613S proteins, SDX detached from the binding site, thereby scoring a large RMSD of 12 and 15 nm (with a log value above 1.1), respectively (Figure 4B and Figure S1 in the Supplementary Materials). However, a unimodal equilibrium was exhibited in I431V, A437G, K540E, K540E/A581G, and A437G/A581G (RMSD in the range of 0.03–1.5 nm), compared with the WT. Although a single conformational dynamic was exhibited by the haplotype A437G/A581G, SDX shifted from its original binding region to a nearby site and remained stably bound over the remaining simulation time. For mutant A613S, SDX remained unstable throughout the simulation, as seen from the multimodal distribution pattern exhibited (Figure 4B). A peculiar, slight flip of the hydroxyl tail of SDX in haplotype A437G/A581G/A613S resulted in a more widely spread bimodal conformational distribution.

### 2.7. Impact of Mutations on the Protein Backbone, Using C-Alpha RMSD

**Figure 5:**
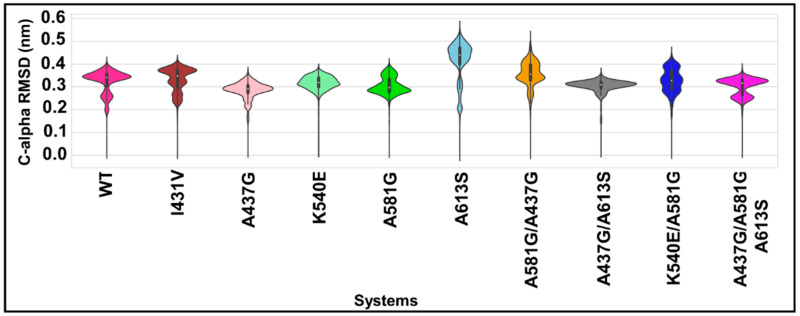
Violin plots of the protein backbone RMSD values across WT (in pink) and mutant *Pf*dhps systems; the interquartile (25th and 75th) is indicated in the black box, with the median in the white dot inside the kernel density plot.

To evaluate the impact of mutations on the stability of the protein backbone, the C-alpha RMSD was calculated. Figure S2 from the Supplementary Materials and Figure 5 show the line trajectory plot versus the simulation time and the kernel density distribution of the C-alpha RMSD values, respectively. From the resultant plots, reliable trajectories were observed for the analyses as all systems exhibited well-equilibrated backbone patterns, except for A437G/A581G and A613G, the positions of which had a higher jump at approximately 40 to 100 ns (Figure S2 of the Supplementary Materials). Regarding backbone motion, the WT exhibited a bimodal distribution pattern with an RMSD of approximately 0.35 nm. In the presence of mutations, single (unimodal) conformational changes were observed for the mutations A437G, K540E, and A437G/A613G (with an RMSD between 0.30 and 0.31 nm). On the other hand, a multiple conformational equilibrium was seen for the mutations I341V and A581G and haplotypes A581G/A613S, A437G/A581G, and A437G/A581G/A613S.

### 2.8. Impact of Mutations on Protein Compactness

**Figure 6:**
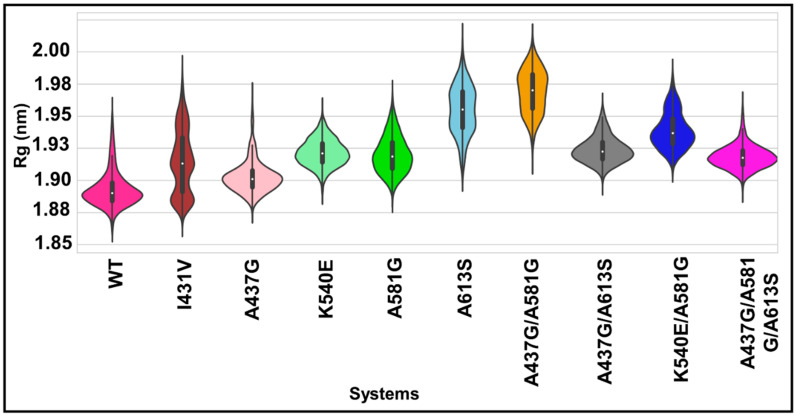
Distribution plot of the Rg values of WT and mutant *Pf*dhps systems. The interquartile ranges (25th and 75th) are represented in the black box, with the median in the white dot inside the kernel density plot.

Shown in Figure 6 is the compactness of proteins in the presence and absence of mutations. It can be seen from the results that the WT protein exhibits a more compact structure, as indicated by the unimodal distribution and lower radius of gyration (Rg) of 1.89 nm. On the other hand, all mutants exhibited a slightly higher Rg (1.90–1.96 nm) compared with WT. Remarkably, a noticeable multimodal equilibrium was attained for mutant I431V, whereas a bimodal conformational distribution was seen for the mutant A613S and the haplotypes K540E/A581G and A437G/A581G.

### 2.9. Effect of Mutations on Per-Residue Fluctuation

**Figure 7:**
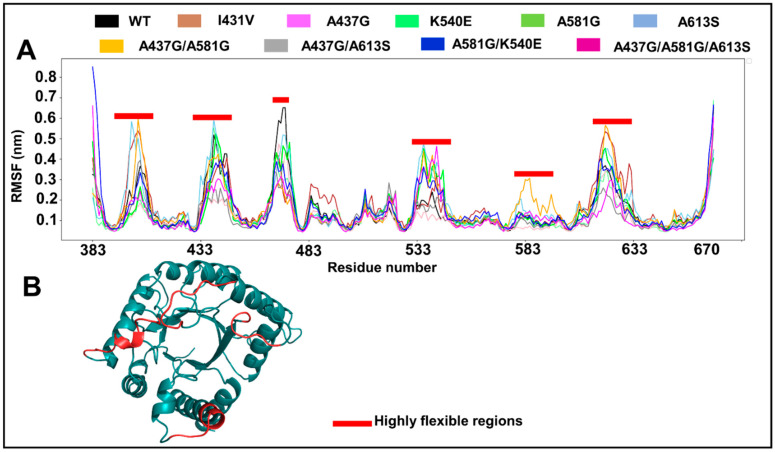
Line and structural mapping plots, showing the fluctuation of residues. (**A**) Line plot indicating the flexible regions between the WT and mutant systems in the presence of SDX. Red bars indicate highly flexible regions. (**B**) Cartoon representation of *Pf*dhps, showing highly flexible regions (in red) mapped to the reference WT structure. Residues are renumbered, based on the template numbering in PDB.

The impacts of mutations on the per-residue flexibility dynamics are shown as line plots in Figure 7A and in the highly flexible regions mapped to the structure in Figure 7B. In general, six regions exhibited high flexibility across all systems (root mean square fluctuation (RMSF) above 3.5 nm). These regions contain residues 401–406, 433–444, 469–475, 532–544, 581–587, and 619–628. With the exception of 469–475, all residues exhibited higher flexibility in the mutants compared with WT. The mapping of highly flexible regions to the structure reveals that the majority of flexible regions correspond to the loop structures (residues 401–406, 433–444, 532–535, 540–544, and 619–621) in close proximity to the active site tunnel. However, residues 537–538 and 622–625 are positioned on α-helices that are posterior to the active centers.

## 3. Discussion

Sulfadoxine resistance is a major issue in malaria treatment and management [30]. Mutations conferring drug resistance can be quickly identified via continuous monitoring utilizing molecular markers; this process will help to provide the underlying information for drug policies [31]. In this study, we explored the mutations and haplotypes of *Pf*dhps isolated from West, Central, and East Africa, Southeast Asia, and South America. Herein, we also further predict their potential effect on protein structure, which could aid in approaches to designing novel drugs by revealing the mechanisms involved in resistance.

The most prevalent *Pf*dhps mutations found were I431V, A437G, K540E/N, A581G, and A613S/T. A high prevalence of the *Pf*dhps A437G mutation was observed across all countries, while *P. falciparum* samples from the EAF population showed the presence of the K540E mutation (Table 1). These mutations were additionally related to SDX resistance, whereas A437G selectivity during IPTp had previously been observed [32,33]. Various reports indicate that A437G has nearly reached saturation in the majority of African locations [34,35]. The levels of A581G are significantly higher in the SEA region, followed by EAF (Malawi and Tanzania), CAF (Cameroon and DR Congo), and WAF (Ghana). Several studies indicate that the prevalence of K540E is low in Central and Western Africa [15,36,37,38]. The *Pf*dhps A581G and A613S/T mutations were previously reported at a low frequency in WAF and EAF, but their prevalence is reported to be rapidly rising in Kenya and Uganda [15,39]. Furthermore, the higher prevalence of K540E and A581G, which form the full (quintuple) and super (sextuple)-SP-resistant haplotypes, with the *Pf*dhfr mutation (N51I, C59R, S108N) and *Pf*dhps mutation (A437G) in EAF and SEA, indicates that resistance originated in these areas and gradually spread to the other endemic areas. This may have been due to the earlier use of SP in these areas than in other endemic areas [40]. The increased prevalence of I431V seen in Cameroon (19%) and Ghana (1.4%) warrants continuous close monitoring. In 2015, a 9.8% prevalence of I431V was reported in Cameroon [15].

With regard to the haplotype analysis, the combination of highly established resistance markers, i.e., A437G, K540E, A581G, and A613S, that are present on the same haplotype indicates that these mutations are not being individually selected. We identified a moderately high prevalence of the triple mutant haplotype GKGS (A437G/A581G/A613S) in the WAF and CAF regions. This combination (GKGS) may be responsible for conferring moderate to increased SP tolerance in these regions.

As a result of missing residues in the WT *Pf*dhps structure, homology modeling was deployed to remodel the structure. The resolution of the template (2.50 Å) indicates a clear density profile. The model evaluation using ProSA showed that the modeled structure is comparable to experimentally determined X-ray structures in PDB. The structure assessment by VERIFY3D revealed that most residues had averaged 3D–1D scores ≥0.2. Stereochemical checks using PROCHECK achieved an acceptable range, indicating the good quality of the modeled structure. Overall, the assessment of the modeled structure using ProSA, PROCHECK, and Verify3D indicated that the generated structures were of good quality and are therefore reliable for use in further structure-based studies. The observed changes in the physicochemical properties of mutations (changes from small to large amino acids and vice versa) could affect the active site architecture and, thus, the high affinity of SDX binding. Following the results of several studies, it has been postulated that small changes to its core amino acids can alter a protein’s structural conformation, enough to destroy a binding site on the surface, leading to a reduced binding affinity for SDX [41]. The mapping of mutations revealed the close proximity of I431V, A437G, K540E, A581G, and A613S to the active tunnel, and that their ability to cause destabilization or stabilization could result in changes to SDX binding. The destabilization of protein active sites in mutated sequences could result in changes such as fluctuations in temperature or pH, which may lead to the loss of protein structure integrity (denature) and its enzymatic ability. Changes in the Gibbs free energy of mutations I431V, A437G, K540E, A581G, A613S, A437G/A581G, and A437G/A581G/A613S revealed a destabilization effect around the regions of the active site resulting from the loss of hydrogen bonds in neighboring residues. The destabilization effect may suggest conformational changes, which could affect SDX binding [42,43].

SDX assumed an optimal orientation at the binding site via molecular docking, interacting with crucial substrate-binding amino acids. Molecular docking employs force-fields and knowledge-based statistical and empirical scoring functions to provide a perspective strength of ligand–protein interactions (i.e., binding energies) [29]. The observed increased or decreased binding energies/modes of SDX in WT and mutant systems are likely to be the result of mutation-induced changes to the active site. Although there were differences in binding energies between WT and mutant systems, a significant difference in binding affinity between WT and the mutant I431V (3.1 kcal/mol) might reveal the decreased binding affinity of SDX to *Pf*dhps, and, thus, could contribute to SP resistance. The decreased protein–ligand binding energy scores and molecular interactions, such as H-bonding in the presence of mutations I431V, K540E, A437G/A581G, K540E/A581G, and A437G/A581G/A613S, may reflect reduced molecular recognition and binding compared with WT. The greater decrease in binding affinity exhibited by mutant I431V protein, compared with WT *Pf*dhps, suggests that the change affects SDX binding.

During MD simulations with the WT system, SDX attained a relatively bimodal conformation, indicating a more stable orientation unique for catalysis. The release of SDX during the early stages of the simulations in mutant A581G and A613S suggests a novel mechanism of action that is adapted to promote resistance. Interestingly, simulation of the mechanism of action has been previously reported for other mutations in *Mycobacterium tuberculosis*, implicated in resistance [44]. A more rigid conformation exhibited by mutants and haplotypes A437G, K540E, A437G/A581G, and K540E/A581G could limit SDX flexibility, which is crucial for catalysis. A multimodal shift by mutant A437G/A613S indicates that there are several positions of equilibrium.

The protein backbone RMSD is the simulation-based measurement of the effect of mutations on protein stability. The multiple conformations seen for the mutations I431V, A437G, A613S, and A581G, and the haplotypes A437G/A581G, A581G/K540E, and A437G/A581G/A613S suggest a higher level of protein backbone instability. Rg represents the compactness of proteins both with and without mutations. The more compact structure exhibited by the WT protein is vital for catalysis. On the other hand, the slightly higher Rg exhibited by all mutants is indicative of increased protein unfolding. The noticeable bimodal conformational distribution for mutations A613S, A437G/A581G, and K450E/A581G and the multimodal distribution for mutant I431V might indicate a high degree of denaturation [44,45,46].

Overall, the differences between the WT and mutant systems are the basis for the mechanism of resistance, promoting protein fitness in the populations. Although SP plays an effective role in preventing malaria in vulnerable populations [32], it is necessary to pay closer attention to the profiles of these mutations to ensure the sustainability of SP for IPTp/c.

## 4. Materials and Methods

### 4.1. Plasmodium Falciparum Sequence Acquisition and Analysis

#### 4.1.1. Study Data Retrieval and Preprocessing

Genomic sequences from 29 countries, spanning West, Central, and East Africa, Southeast Asia, and South America, were selected from the MalariaGEN *Plasmodium falciparum* (Pf3k) Community Project (release version 6 of the database) [24] in variant call format (VCF). Gene variants on chromosome 8 were extracted. Additionally, individual-level data collected from Begoro and the Cape Coast in the forest and coastal ecological zones of Ghana over 4 years (2014–2017) were included [23]. These data consisted of 150 and 181 genomic sequences from Begoro and the Cape Coast, respectively. *Pf*dhps genetic variants on chromosome 8 (position: 547,896–551,057) were extracted for all populations using BCFtools version 1.9 and in-house Python scripts. Prior to variant extraction, biallelic single-nucleotide polymorphisms (SNPs) obtained for all populations were quality controlled using the following rules: only SNPs that passed all VCF filters were maintained; isolates with >10% of missing SNPs were removed; SNPs with >5% of missing SNP data were removed, using PLINK v1.9 [47]. The SNPs that remained were imputed and phased using Beagle v5.1. The extracted *Pf*dhps sequences were then translated into amino acids, using the in-house Python script.

#### 4.1.2. Sequence Data Analyses and Statistics

The sequence data for *Pf*dhps were first analyzed for SNPs (A437G, A581G, A613S/T, K540E/N/Y) and defined as either wild-type (WT)—“isolate with no mutation detected” or mutant—“isolate with mutation detected”. Subsequently, haplotypes for the *Pf*dhps were constructed and grouped as follows: WT—“isolate with no mutation detected” or either single, double, triple, or quadruple mutants for isolate with 1, 2, 3, or 4 mutant alleles, respectively. The prevalence of mutations was estimated as the proportion of isolates with a mutant allele among the total number of successfully analyzed isolates. The prevalence of mutations and haplotypes in *Pf*dhps, i.e., I431V, A437G, A581G, A613S/T, and K540E/N/Y, were then estimated for each country. The differences in the prevalence of drug-resistant alleles and haplotypes among countries were assessed using Chi-square (χ^2^) and/or Fisher’s exact tests. The STATA software package, version 12 (StataCorp LP, College Station, TX, USA), was used to perform the statistical analyses. Statistical significance was inferred for *p*-values < 0.05.

### 4.2. Structure-Based Analysis

#### 4.2.1. Wild-Type and Mutant Structure Retrieval and Assessment

The WT three-dimensional (3D) structure of *Pf*dhps-HPPK (PDB ID: 6JWQ) was obtained from the Protein Data Bank (PDB) [28]. As a result of the presence of missing residues in the available structure, homology modeling was employed to remodel the full-length protein structure using the MODELLER (version 9.18) tool [48]. Initially, the protein interactive modeling (PRI-MO) protein structure prediction server was used to select a suitable template, based on the highest sequence identity and query coverage to the target sequence [49]. All crystalized water molecules and unwanted ligands were removed from the structure. Additionally, the PROCHECK [27], Verify3D [26], and ProSA [25] tools were utilized to further authenticate the template. Considering the listed criteria, the sequence of *Pf*dhps was extracted from PlasmoDB. The template–target alignment from MAFFT [50] was employed to generate “pir” files for modeling. A total of 100 models were generated using a “very slow refinement” molecular dynamics level. The optimal model was chosen by rating all generated models using the normalized discrete optimized potential energy (z-DOPE) scoring profile [51] and validating the three models with the highest scores, as per the template. A consensus result of the different validation tools was evaluated to select the best model. Additionally, the protein structure of mutations was manually inserted at their appropriate residue positions, using the Discovery Studio (DS) visualization tools [52]. DS was employed to minimize the possible structural variations in mutant structures.

#### 4.2.2. Mutation Mapping and Molecular Docking

The identified mutations were mapped to the generated structure, and other physiochemical property changes in the amino acid residues and the effect of mutations on the protein were evaluated using PyMOL [53] and the *DynaMut* tool [54], respectively. The *DynaMut* tool evaluates the impact of mutations on protein stability and dynamics. A positive score indicates a destabilization effect, while a negative score represents a stabilization effect. To predict the binding effect of SDX across mutant and wild-type *Pf*dhps proteins, molecular docking was performed using AutoDock Vina. Prior to docking, the 3D structure of SDX was retrieved from DrugBank (ID: DB01299) [55] in SMILES format. The chemical compound was constructed and minimized to the lowest energy geometry, using RDKit [56]. Initial docking validation was conducted to evaluate the consistency of the AutoDock Vina docking poses. The protein and ligand *pdbqt* input files were generated using AutoDockTools 1.5.6 (ADT) [57], where all nonpolar hydrogens were fused and partial charges were assigned using the Gasteiger–Huckel method. Initially, each protein was subjected to blind docking simulations with 320 exhaustiveness, using a cuboid box with a diameter of 120 × 120 × 120 and a grid spacing of 0.375. The intermolecular interactions between SDX and each protein were determined using LigPlot+ [58] and DS 2D plots.

#### 4.2.3. All-Atom Molecular Dynamics Simulations of *Pf*dhps WT and Mutant Proteins

A total of 150 ns all-atom molecular dynamics (MD) simulations were conducted using GROMACS (version 2019) [59] for *Pf*dhps WT and mutant proteins with SDX compounds, bound to the active site. The AMBER03 force field [60] and the ACPYPE tool [61] were used to create input files for the structure and the ligand topology, respectively, which were GROMACS-compatible. A total of nine systems (WT) and eight mutant systems (I431V, A437G, K540E, A581G, A613S, A437G/A613S, A437G/A581G, K540E/A581G, and A437G/A581G/A613S) were solvated in a cubic box, with a minimal gap of 1 Å between the box edge and the protein, using the TIP3P water model [62]. Then, 0.15 M NaCl was used to neutralize all systems. The relaxed systems converged to a maximum force of 1000 kJ/mol/nm after the solved systems had been initially minimized for 5000 steps, using the steepest descent algorithm. Following minimization, systems were equilibrated at a constant number, volume, and temperature (NVT) using the modified Berendsen thermostat algorithm at a 300 K temperature and constant volume [63], and then at NPT (constant number of particles, pressure, and temperature) using the Parrinello–Rahman barostat algorithm at 1 bar pressure and at a constant volume and temperature [64]. Systems coupling groups and time restrictions were defined in each ensemble at fs. The LINCS holonomic constraints algorithm [65] was used to restrict all bonds; however, the particle-mesh Ewald (PME) algorithm [66] was configured to take long-range electrostatic interactions into account. The Center for High-Performance Computing (CHPC), Cape Town, South Africa, was used to implement the overall MD protocol. After every 10 ps, structural coordinates were written, and periodic boundary conditions (PBC) were eliminated. Post-MD analyses, such as ligand and C-alpha RMSD, RMSF, and Rg analyses, were performed.

## 5. Conclusions

Here, the prevalence of circulating SDX-resistant *Pf*dhps mutations was determined, and their effects on protein functionality were revealed via computational approaches, including SNP analysis, energy-based analysis, molecular docking, and MD simulation. The prevalence of mutants and their corresponding haplotype prevalence were assessed across Africa and other regions. The analysis indicated that the mutation A613S had variable prevalence across most of the studied regions, except for isolates from DR Congo and Malawi. Molecular interactions from docking revealed a loss of interactions in mutants relative to the WT protein. Interestingly, SDX formed unfavorable bonds with the residue His586 in the mutant K540E and haplotype A437G/A613S. The ligand RMSD in the MD simulations indicated that SDX dissociates from the active site of mutants A581G and A613S before the end of the simulation run time. SDX remained unstable in mutants I431V, A613S, and A437G/A581G/A613S. The global protein RMSD indicated several examples of conformational dynamics in mutants with I431V, A581G, A613S, A437G/A581S, A581G/K540E, and A437G/A581G/A613S. Additionally, Rg revealed that mutations I431V, A613S, A437G/A581G, and K540E/A581G are associated with protein unfolding behavior.

Overall, continuous analysis of *Pf*dhps SNPs is encouraged to track mutations. The molecular effects of SNPs on the structure of *Pf*dhps could cause the loss of protein function. These findings may have implications for drug deployment for IPTp/c and the mechanisms of drug resistance and should be monitored. Further interaction studies with mutated proteins expressed in the presence of SDX should be conducted to provide insight into the mechanism of protein destabilization and the degree of loss of protein function, due to destabilization.

## Figures and Tables

**Table 1:** Global prevalence of missense mutations detected on *Pf*dhps.

Region	Countries	Mutations, % (*n/N*)						
		A437G	K540E	K540N	A581G	A613S	A613T	I431V
WAF	Ghana	94.94 (1352/1424)	2.4 (33/1374)	0.07 (1/1374)	2.18 (30/1374)	14.3 (197/1376)	0 (0/1376)	1.4 (2/1374)
	Gambia	74.64 (309/414)	3.12 (13/417)	0 (0/417)	0 (0/421)	6.19 (26/420)	0 (0/420)	0 (420)
	Mali	55.38 (247/446)	0.89 (4/447)	0 (0/447)	0.22 (1/447)	9.17 (41/447)	0 (0/447)	0 (0/447)
CAF	Cameroon	95.8 (228/238)	0 (0/239)	0.42 (1/239)	22.36 (53/237)	29.11 (69/237)	0 (0/237)	19 (46/237)
	DR Congo	96.72 (354/366)	11.75 (43/366)	0 (0/366)	3.55 (13/366)	0 (0/366)	0 (0/366)	0 (0/366)
EAF	Kenya	93.55 (116/124)	87.1 (108/124)	0 (0/124)	0.8 (1/125)	1.59 (2/126)	2.38 (3/126)	0 (0/124)
	Tanzania	90.77 (305/336)	88.46 (299/338)	0 (0/338)	28.78 (97/337)	0.89 (3/337)	0 (0/337)	0 (0/337)
	Malawi	100 (257/257)	99.61 (257/258)	0 (0/258)	4.65 (12/258)	0 (0/258)	0 (0/258)	0 (0/258)
SEA	Thailand	99.79 (959/961)	91.53 (865/945)	6.88 (65/945)	81.59 (780/956)	0.1 (1/962)	0.42 (4/962)	0 (0/956)
	Cambodia	93.03 (1055/1134)	37.46 (418/1116)	37.37 (417/1116)	44.36 (503/1134)	0.44 (5/1135)	0.53 (6/1135)	0 (0/1135)
	Vietnam	85.6 (214/250)	41.9 (106/253)	4.35 (11/253)	14.57 (37/254)	16.54 (42/254)	0 (0/254)	0 (0/254)
SAM	Colombia	17.65 (3/17)	0 (0/17)	0 (0/17)	0 (0/17)	5.88 (1/17)	0 (0/17)	0 (0/17)
	Peru	55.17 (16/29)	13.79 (4/29)	0 (0/29)	31.03 (9/29)	17.24 (5/29)	0 (0/29)	0 (0/29)

WAF: West Africa; CAF: Central Africa; EAF: East Africa; SEA: Southeast Asia; SAM: South America.

**Table 2:** Global frequency of key *Pf*dhps haplotypes. Amino acid changes are highlighted in bold.

	WAF			CAF		EAF			SEA			SAM	
	Ghana % (*n/N*)	Gambia % (*n/N*)	Mali % (*n/N*)	Cameroon % (*n/N*)	DR Congo % (*n/N*)	Kenya % (*n/N*)	Tanzania % (*n/N*)	Malawi % (*n/N*)	Thailand % (*n/N*)	Cambodia % (*n/N*)	Vietnam % (*n/N*)	Colombia % (*n/N*)	Peru % (*n/N*)
A437K540A581 A613	4.25 (58/1366)	23.95 (97/405)	42.60 (190/446)	3.80 (9/237)	3.28 (12/366)	5.69 (7/123)	8.90 (29/326)	0 (0/257)	0.21 (2/935)	7.03 (77/1095)	13.93 (34/244)	82.35 (14/17)	44.83 (13/29)
A437K540A581 **S613**	0.81 (11/1366)	0.99 (4/405)	2.02 (9/446)	0 (0/237)	0 (0/366)	0 (0/123)	0.61 (2/326)	0 (0/257)	0 (0/935)	0.09 (1/1095)	0 (0/244)	0 (0/17)	0 (0/29)
A437K540**G581** **S613**	0 (0/1366)	0 (0/405)	0 (0/446)	0.42 (1/237)	0 (0/366)	0 (0/123)	0 (0/326)	0 (0/257)	0 (0/935)	0 (0/1095)	0 (0/244)	0 (0/17)	0 (0/29)
A437**E540**A581 A613	0 (0/1366)	0 (0/405)	0 (0/446)	0 (0/237)	0 (0/366)	0.81 (1/123)	0 (0/326)	0 (0/257)	0 (0/935)	0 (0/1095)	0.82 (2/244)	0 (0/17)	0 (0/29)
**G437E540**A581 A613	2.27 (31/1366)	2.96 (12/405)	0.67 (3/446)	0 (0/237)	8.47 (31/366)	85.37 (105/123)	59.51 (194/326)	94.94 (244/257)	17.43 (163/935)	32.97 (361/1095)	17.21 (42/244)	0 (0/17)	0 (0/29)
**G437E540**A581 **S613**	0.07 (1/1366)	0.25 (1/405)	0.22 (1/446)	0 (0/237)	0 (0/366)	0 (0/123)	0.31 (1/326)	0 (0/257)	0.11 (1/935)	0.18 (2/1095)	16.39 (40/244)	0 (0/17)	0 (0/29)
**G437E540**A581 **T613**	0 (0/1366)	0 (0/405)	0 (0/446)	0 (0/237)	0 (0/366)	0 (0/123)	0 (0/326)	0 (0/257)	0.43 (4/935)	0.18 (2/1095)	0 (0/244)	0 (0/17)	0 (0/29)
**G437E540G581** A613	0.07 (1/1366)	0 (0/405)	0 (0/446)	0 (0/237)	3.28 (12/366)	0.81 (1/123)	29.45 (96/326)	4.67 (12/257)	73.69 (689/935)	4.38 (48/1095)	7.38 (18/244)	0 (0/17)	13.79 (4/29)
**G437**K540A581 A613	79.06 (1080/1366)	66.67 (270/405)	47.53 (212/446)	66.67 (158/237)	84.70 (310/366)	3.25 (4/123)	1.23 (4/326)	0.39 (1/257)	0 (0/935)	15.16 (166/1095)	38.11 (93/244)	11.76 (2/17)	6.90 (2/29)
**G437**K540A581 **S613**	11.35 (155/1366)	5.19 (21/405)	6.73 (30/446)	7.17 (17/237)	0 (0/366)	1.63 (2/123)	0 (0/326)	0 (0/257)	0 (0/935)	0.09 (1/1095)	0 (0/244)	5.88 (1/17)	17.24 (5/29)
**G437**K540A581 **T613**	0 (0/1366)	0 (0/405)	0 (0/446)	0 (0/237)	0 (0/366)	2.44 (3/123)	0 (0/326)	0 (0/257)	0 (0/935)	0.37 (4/1095)	0 (0/244)	0 (0/17)	0 (0/29)
**G437**K540**G581** A613	0 (0/1366)	0 (0/405)	0 (0/446)	0 (0/237)	0.27 (1/366)	0 (0/123)	0 (0/326)	0 (0/257)	1.28 (12/935)	2.01 (22/1095)	1.64 (4/244)	0 (0/17)	17.24 (5/29)
**G437**K540**G581** **S613**	2.05 (28/1366)	0 (0/405)	0.22 (1/446)	21.52 (51/237)	0 (0/366)	0 (0/123)	0 (0/326)	0 (0/257)	0 (0/935)	0 (0/1095)	0 (0/244)	0 (0/17)	0 (0/29)
**G437N540**A581 A613	0 (0/1366)	0 (0/405)	0 (0/446)	0 (0/237)	0 (0/366)	0 (0/123)	0 (0/326)	0 (0/257)	0.21 (2/935)	0.09 (1/1095)	0 (0/244)	0 (0/17)	0 (0/29)
**G437N540G581** A613	0 (0/1366)	0 (0/405)	0 (0/446)	0.42 (1/237)	0 (0/366)	0 (0/123)	0 (0/326)	0 (0/257)	6.63 (62/935)	37.35 (409/1095)	4.51 (11/244)	0 (0/17)	0 (0/29)
**G437N54**0**G581** **S613**	0.07 (1/1366)	0 (0/405)	0 (0/446)	0 (0/237)	0 (0/366)	0 (0/123)	0 (0/326)	0 (0/257)	0 (0/935)	0 (0/1095)	0 (0/244)	0 (0/17)	0 (0/29)
**G437Y540**A581 A613	0 (0/1366)	0 (0/405)	0 (0/446)	0 (0/237)	0 (0/366)	0 (0/123)	0 (0/326)	0 (0/257)	0 (0/935)	0.09 (1/1095)	0 (0/244)	0 (0/17)	0 (0/29)

**Table 3:** Identified changes in the amino acid properties.

Mutation	Physiochemical Changes	Location on Structure	Amino Acid Changes	Mutation Effect	Entropy Score
I431V	Hydrophobic to hydrophobic	Buried	Large to small	Destabilizing	0.39
A437G	Hydrophobic to hydrophobic	Surface	Large to small	Destabilizing	0.66
K540E	Basic to polar	Surface	Small to large	Destabilizing	0.37
A581G	Hydrophobic to hydrophobic	Buried	Large to small	Destabilizing	0.31
A613S	Hydrophobic to polar	Surface	Small to large	Destabilizing	0.31
A437**G**/A581**G**				Destabilizing	0.41
A437**G**/A613**S**				Stabilizing	−0.06
A437**G**/A581**G**/A613**S**				Destabilizing	0.44

## Data Availability

Data will be made available upon request.

## Supplementary Materials

The following supporting information can be downloaded at: https://www.mdpi.com/article/10.3390/molecules28010145/s1. Figure S1: Kernel distribution plot showing the ligand RMSD scores occurring in WT and mutant proteins. The white dots represent the median, whereas the thick black bars in the centers illustrate the interquartile range. (A) WT (pink) and mutant systems where SDX was retained during the 150 ns simulation. (B) Mutant proteins where SDX was released before the simulation run time, compared to the WT (pink). The same WT system is represented on each panel but on a different scale; Figure S2: Line plot of protein RMSD showing protein stability indices; Table S1: Breakdown summary of analysis set samples organized by geography. Regions are categorized into five regions. These comprised East Africa (EAF), West Africa (WAF), Central Africa (CAF), Southeast Asia (SEA) and Southern America (SAM).

## Sample Availability

Sample of the compound are available from the authors.
