# Supplementary material for: Global Analysis of Plasmodium falciparum Dihydropteroate Synthase Variants Associated with Sulfadoxine Resistance Reveals Variant Distribution and Mechanisms of Resistance: A Computational-Based Study
Source: Molecules. 2022 Dec 24;28(1):145. doi: 10.3390/molecules28010145 (PMC9822128; doi:10.3390/molecules28010145)
Supplement: Supplementary file 1 [file molecules-28-00145-s001.zip › molecules-2083066-supplementary.pdf]

Supplementary Materials

Global Analysis of *Plasmodium falciparum* Dihydropteroate Synthase Variants Associated with Sulfadoxine Resistance Reveals Variant Distribution and Mechanisms of Resistance: A Computational-Based Study

Rita Afriyie Boateng [1], James L. Myers-Hansen [1], Nigel N. O. Dolling [1], Benedicta A. Mensah [1], Elia Brodsky [2], Mohit Mazumder [2] and Anita Ghansah [1,*]

[1] Noguchi Memorial Institute for Medical Research, College of Health Sciences, University of Ghana, Legon, Accra P.O. Box LG 581, Ghana
[2] Pine Biotech, Inc., 1441 Canal St., New Orleans, LA 70112, USA
* Correspondence: aghansah@noguchi.ug.edu.gh; Tel.: +23-327-145-535

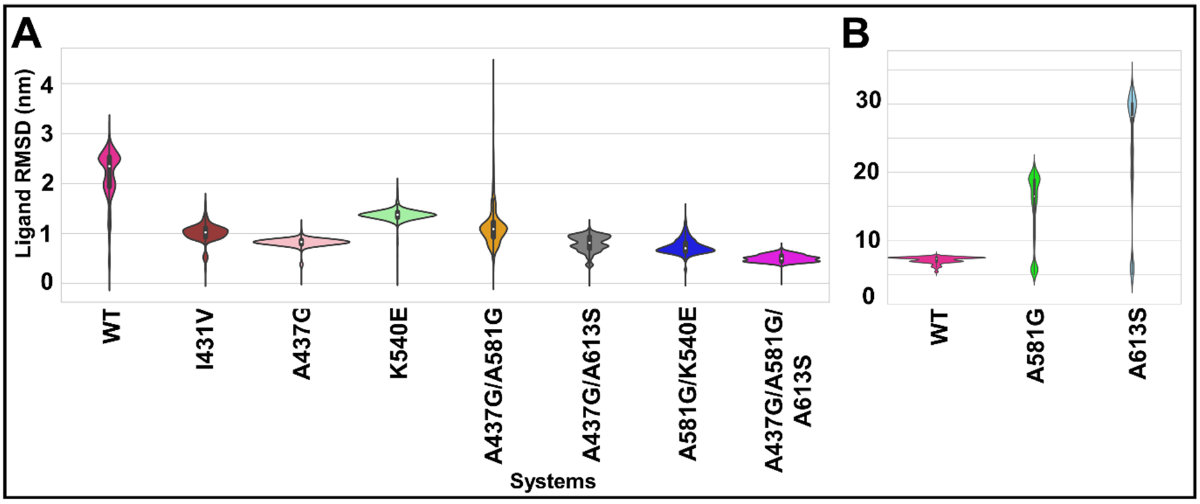

Figure S1. Kernel distribution plot showing ligand RMSD scores occurring in WT and mutant proteins. The white dots represent the median, whereas the thick black bars in the centers illustrate the interquartile range. (A) WT (pink) and mutant systems where SDX was retained during the 150 ns simulation. (B) Mutant proteins where SDX was released before the simulation run time compared to the WT (pink). The same WT system is represented on each panel but on a different scale.

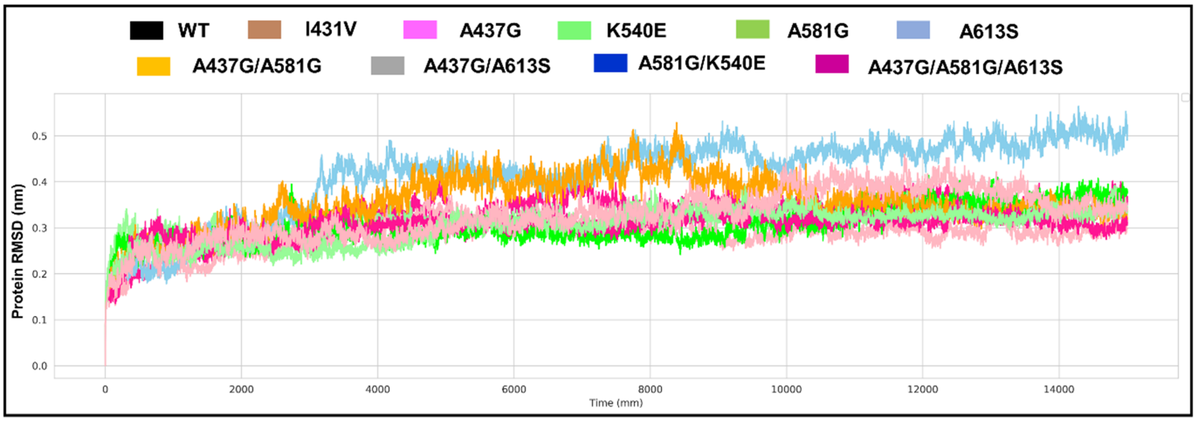

Figure S2. Line plot of protein RMSD showing protein stability indices.

Table S1. Breakdown summary of analysis set samples organized by geography. Regions are categorized into five regions. These comprised East Africa (EAF), West Africa (WAF), Central Africa (CAF), Southeast Asia (SEA) and Southern America (SAM).

Region	Country	Samples	Region	Country	Samples
WAF	Ghana	1432	SEA	Cambodia	1156
	Gambia	430		Thailand	965
	Mali	448		Vietnam	260
CAF	Cameroon	239			
	DR Congo	366			
EAF	Kenya	126	SAM	Columbia	17
	Tanzania	344		Peru	295
	Malawi	258			
**Total**			**6336**		
